# Genome Sequence of the Psychrophilic Bacterium *Tenacibaculum ovolyticum* Strain da5A-8 Isolated from Deep Seawater

**DOI:** 10.1128/genomeA.00644-16

**Published:** 2016-06-30

**Authors:** Maki Teramoto, Zhenyu Zhai, Ayumi Komatsu, Keigo Shibayama, Masato Suzuki

**Affiliations:** a Oceanography Section, Kochi University, Kochi, Japan; b Department of Bacteriology II, National Institute of Infectious Diseases, Tokyo, Japan

## Abstract

Some bacterial species of the genus *Tenacibaculum*, including *Tenacibaculum ovolyticum*, have been known as fish pathogens in the sea. So far, the only published genome sequence for this genus is for *Tenacibaculum dicentrarchi*, which could also be a fish pathogen. Strain da5A-8, showing 100% identity to the 16S rRNA gene sequence of *T. ovolyticum* DSM 18103^T^, was isolated from seawater at a depth of 344 m in Kochi, Japan, and grew optimally at 10 to 20°C. The genome sequence of strain da5A-8 revealed the possible virulence genes commonly observed in the genus *Tenacibaculum*.

## GENOME ANNOUNCEMENT

The genus *Tenacibaculum* currently comprises 20 species of marine origin (http://www.bacterio.net/tenacibaculum.html). In this genus, *T. maritimum* (1), *T. ovolyticum* (2, 3), *T. discolor*, and *T. gallaicum* (4, 5) are fish pathogens. *T. soleae* (6) and *T. dicentrarchi* (7, 8) from diseased fish could also be pathogens. So far, the genome sequence has been published only for *T. dicentrarchi* AY7486TD, isolated from a diseased salmon (8).

A bacterial strain, designated da5A-8, was isolated from seawater from a depth of 344 m in Kochi, Japan (33°18′N, 134°13′E), in January 2013. The 16S rRNA gene sequence (accession number LC144619) showed 100% identity to that of *T. ovolyticum* DSM 18103^T^ (NR_040912), indicating that strain da5A-8 belongs to *T. ovolyticum*.

*T. ovolyticum* has been shown to be abundant, 4.5% of the total bacteria, at a depth of 320 m (at 1 to 2°C; Argo JAMSTEC) in the Japan Sea Proper Water, while *Tenacibaculum* spp. other than *T. ovolyticum* constituted <0.1% of the total bacteria in the water (unpublished data). Also, *T. ovolyticum* has not been detected from surface seawater in Japan (unpublished data). Consistently, strain da5A-8 was psychrophilic: it grew at 4, 10, 15, 20, and 25°C on dR2A-SW plates (9), growing optimally at 10 to 20°C. Other *Tenacibaculum* spp. grow optimally at a temperature of 25°C or higher (http://www.bacterio.net/tenacibaculum.html). Another characteristic of the Japan Sea Proper Water with a characteristic of low temperature is that it can harbor a large amount of eggs and larvae of Atlantic halibut, for which *T. ovolyticum* is a pathogen (2, 3). Proteolytic activity of *T. ovolyticum* dissolves the egg shell, which could lead to the death of the embryo (2, 3).

To examine the virulence-associated genes in strain da5A-8, whole-genome shotgun sequencing was performed using the Illumina HiSeq 2500 pyrosequencing platform (500- to 750-bp insert size). Paired-end reads (2 × 150-bp) were assembled *de novo* using CLC Genomics Workbench version 8.5 (Qiagen). The draft genome sequence consisted of 282 contigs, yielding a total sequence of 4,148,120 bp with an *N*_50_ contig size of 44,580 bp. The mean G+C content was 29.5%. A total of 3,969 coding DNA sequences were annotated by RASTtk (http://rast.nmpdr.org). One copy of the 16S rRNA gene was present, showing 95.7 to 96.6% sequence identity to the genes (9 copies) in strain AY7486TD (8).

Genes encoding metallopeptidases and hemolysins were detected in strain da5A-8 by RASTtk. Gene clusters encoding structural components of the type IX secretion system (T9SS) were detected by RASTtk and also by TXSScan (http://mobyle.pasteur.fr/cgi-bin/portal.py#forms::txsscan). Bacterial pathogens frequently use such a protein secretion system to interact with their hosts. T9SS is found in 61.9% of the species of the phylum *Bacteroidetes* (10) and is required for gliding motility and secretion of the surface adhesins. The detected genes are also found in strain AY7486TD (8) and are thus possible virulence genes commonly observed in *Tenacibaculum*. More details of strain da5A-8 will be reported in a future publication.

### Nucleotide sequence accession numbers.

This whole-genome shotgun project has been deposited at DDBJ/EMBL/GenBank under the accession number BDCW00000000. The version described in this paper is the first version, BDCW00000000.1.
